# Establishing a role of the insulin receptor in microglia

**DOI:** 10.4103/NRR.NRR-D-25-00722

**Published:** 2025-08-13

**Authors:** William A. Banks, Elizabeth M. Rhea

**Affiliations:** Geriatric Research, Education, and Clinical Center, Veterans Affairs Puget Sound Health Care System, Seattle, WA, USA; Division of Gerontology and Geriatric Medicine, Department of Medicine, University of Washington, Seattle, WA, USA

**Figure 1:**
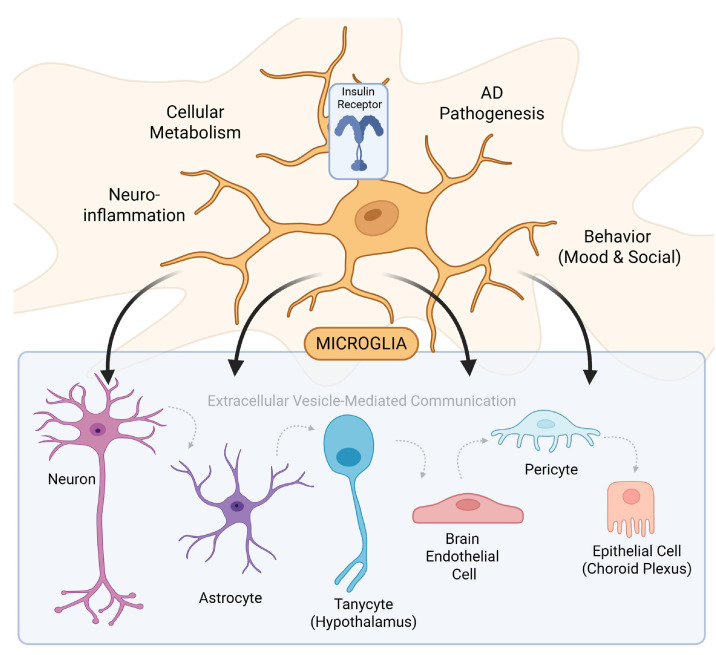
Novel findings for the role of the microglial insulin receptor and proposed future directions. Chen et al. (2025) recently explored the intracellular role of the microglial insulin receptor for the first time and the impact on behavior and Alzheimer’s disease (AD) pathology. Knockdown of the insulin receptor in microglia resulted in expected detrimental changes in cellular metabolism, induction of neuroinflammation, and exacerbated AD pathology. Future work remains to identify the paracrine response to loss of microglial insulin receptor signaling and what the impact may be on other surrounding cell types, such as neurons, astrocytes, pericytes, hypothalamic tanycytes, brain endothelial cells, and epithelial cells (the latter three of which are important for nutrient transfer into the brain). Knowing now that cells can communicate and regulate cell metabolism via extracellular vesicles, trading cargo, a new frontier of research has been opened, particularly within central nervous system-cellular communication. Created with BioRender.com.

Brain insulin resistance (BIR) is a prevalent detrimental feature of Alzheimer’s disease (AD) and all-cause dementia. Therapies designed to activate insulin signaling and enhance insulin receptor sensitivity have proven beneficial for cognitive enhancement in pre-clinical models, non-human primates, and humans. BIR encompasses dysregulated brain insulin signaling, which is either due to insulin receptor resistance, reduced insulin receptor levels, or reduced levels of insulin in the brain, affecting processes involved in AD development and progression. While prior work has shown BIR is a nexus between metabolic dysfunction and neurodegenerative disease, a recent work by Chen et al. (2025) re-emphasizes a role of BIR in AD, highlighting microglia as a key mediator. This recent work is a great step forward in answering many of these questions and shows how central insulin receptor resistance of microglia can result in altered cellular metabolism, neuroinflammation, altered mood and social behavior, and accelerated AD pathogenesis (**Figure 1**).

Both type 1 and type 2 diabetes mellitus are associated with an increased incidence of AD. Both forms of diabetes are associated with micro- and macrovascular changes and episodes of iatrogenic hypoglycemia, any of which can increase the incidence of cognitive dysfunction. AD is associated with decreased transport of glucose across the blood–brain barrier, lower levels of insulin in the cerebrospinal fluid, and insulin receptor resistance at the hippocampus and other brain regions. Previous studies have shown that brain insulin has little effect on blood glucose levels and that resistance at the brain insulin receptor occurs in nearly all AD patients, including the majority who do not have diabetes. As such, the field is in desperate need to understand the cause and consequences of BIR. Here, Chen et al. (2025) studied the consequences of BIR in the key cell type of microglia.

Microglia are the primary resident immune cell of the brain and contribute to amyloid plaque clearance and neuroinflammation regulation in AD. The work by Chen et al. (2025) highlights not only the role of microglia insulin signaling in basal conditions, but also the impact on AD-like neuropathology (**Figure 1**). Novel inducible transgenic mice lacking the insulin receptor in Tmem119^+^ cells, a frequently used marker of microglia, were generated (microglia insulin receptor knockout, MG-IRKO) and bred with the 5×FAD mouse, a widely used transgenic model of AD. Inducible knock-down of the insulin receptor was performed after the developmental period of 6 weeks of age, which is a key consideration given the importance of insulin signaling growth factor properties during development. Another important consideration is that in AD, microglia shift from a basal, resting state (with higher levels of Tmem119^+^ cells) to an activated state (expressing more Iba1). Such a shift in microglial activation was also observed in the study by Chen et al. (2025). As *Tmem119* expression is already decreased in 5×FAD mice (Kiani Shabestari et al., 2022), the results presented by Chen et al. (2025) may be even more dramatic in AD. Reduced levels of the microglial insulin receptor in the 5×FAD mouse model of AD led to exacerbated whole brain microglial activation and increased plaque deposition compared to 5×FAD alone, indicating a regulation of these processes by the microglial insulin receptor. Further, *in vitro* studies in primary microglia identified phagocytosis of amyloid-beta (Aβ) was reduced, likely contributing to the increased plaque deposition observed *in vivo*. Inflammation has long been known to decrease brain efflux of Aβ as well (Jaeger et al., 2009), thus contributing to Aβ accumulation in the brain. The results of Chen et al. (2025) thus support reduced insulin receptor levels in microglia as fostering neuroinflammation-related Aβ retention and plaque formation. Similar data were reported by the same authors in mouse models with reduced insulin receptor levels in astrocytes (Chen et al., 2023), highlighting complementary roles for these two cell types.

In defining the phenotype of the MG-IRKO mouse model, Chen et al. (2025) found minimal effects on peripheral metabolism with reduced levels of the microglial insulin receptor, including the paradoxic effect of slightly increased systemic insulin sensitivity. Such paradoxic effects, like increased blood glucose levels following central nervous system (CNS) administration of insulin, were first shown by Supniewski et al. in 1927. Further, anxiety and working memory were largely unaffected in these baseline conditions, yet there were increased depressive-like behaviors and impairments in social interactions. While prior work has indicated a role for the CNS insulin receptor in these behaviors (Kleinridders and Pothos, 2019; Erichsen et al., 2022), this work for the first time points towards a specific role of microglia. As BIR can be treated with intranasal insulin or anti-diabetic medications such as glucagon-like peptide-1 receptor agonists with minimal serious side effects, it is possible individuals with these conditions may benefit from BIR-targeting therapies.

Using RiboTag, Chen et al. (2025) were able to more precisely measure the mRNA transcripts that predict protein expression. A similar number of transcripts were upregulated compared to downregulated, highlighting the diverse role the insulin receptor has in regulation of CNS genes. MG-IRKO globally reduced genes involved in mitochondrial function and increased genes associated with immune activation. These findings on neuroinflammation are very similar to those found after administration of insulin into the CNS through the intranasal route in a sporadic mouse model of AD (Rhea et al., 2019) and further support roles for insulin signaling in immunometabolism and neuroimmunometabolism (Mitra et al., 2022).

Interestingly, while there are many processes similarly affected between the cell specific IRKO models, it may be even more important to pay attention to the differences, particularly under basal versus diseased conditions. For example, IRKO astrocytes exhibited reduced glycolytic flux, but a higher basal mitochondrial respiration (Garcia-Caceres et al., 2016), while IRKO microglia surprisingly revealed increased glycolysis, but reduced expression of mitochondrial complex genes. These divergent responses illustrate differential roles of the insulin receptor on glycolysis within these two CNS cell types. As this work was performed in *in vitro* models, future work will need to investigate whether these effects are solely intrinsic properties or if extrinsic mechanisms could also be at play. We look forward to reading more about the contrasting cell-specific roles of the insulin receptor by these groups. One rationale for the divergent responses may be particularly important because of the dual nature of insulin resistance. Type 1 diabetes mellitus is primarily caused by a deficiency of insulin and type 2 diabetes mellitus is primarily driven by insulin receptor resistance. However, BIR has both low CSF levels of insulin and impaired responsiveness of the brain insulin receptor. Although either low insulin levels, decreased receptor sensitivity, or reduced insulin receptor levels result in a deficiency of insulin action in the CNS, the different causes may result in different clinical presentations. The blood-to-brain insulin transport rates vary among brain regions, likely resulting in different concentrations of interstitial fluid insulin in the various brain regions, and it should not be assumed that all brain regions have the same degree of insulin resistance. To our knowledge, a whole brain mapping of BIR in human, post-mortem tissue during AD progression, similar to what has been done for amyloid β or phosphorylated tau, has not been completed. Prior work in post-mortem tissue from individuals with AD, without diabetes mellitus, reveals that insulin receptor insensitivity develops in the hippocampal formation to a greater degree than the cerebellar cortex (Talbot et al., 2012). Such brain regional variations in presentation of and sensitivity to insulin could result in different BIR syndromes. This could explain, as suggested by the findings of Chen et al. (2025), how heterogeneity of BIR could be involved in syndromes as different as autism and AD and may be involved in the heterogeneity of AD itself.

Understanding insensitivity as expressed by different cell types is also critically important. Prior work by the Reagan group has shown that reduced hippocampal insulin receptor levels elicit a different phenotype than reduced hypothalamic insulin receptor levels (Erichsen et al., 2022). We now need to combine both lines of work, investigating the effects of regional glial, neuronal, endothelial, and pericyte insulin receptor resistance in the development of disease. The latter two cell types are important given their key roles in maintaining the neurovascular unit, the primary structure regulating insulin entry into the brain and blood–brain barrier integrity. As pericytes are implicated in diabetes-induced blood-brain barrier disruption, whether loss of pericytes, and therefore loss of the pericyte insulin receptor, leads to dysregulation in other CNS-cell types remains to be determined.

As such, these transgenic models with cell-specific insulin receptor knockdown may aid in understanding the etiology of BIR and the cell types initially affected. It is currently unclear which CNS-cell type may first exhibit insulin resistance in the development of AD and whether there is a detrimental cascading effect on other CNS-cell types, initiating insulin resistance (**Figure 1**). Understanding the initial cascade of events leading to global BIR will provide a therapeutic starting point early in the disease.
